# Preoperative Local Infiltration of Tranexamic Acid in Posterior Lumbar Fusion: A Prospective Controlled Study

**DOI:** 10.7759/cureus.108333

**Published:** 2026-05-06

**Authors:** Likith Srinivas, Amarnath Dasari, Anirudh S Rao, Sanjay KG, Aravind Devendrappa, Mohammed Shahid

**Affiliations:** 1 Orthopedics, Sanjay Gandhi Hospital, Bangalore, IND; 2 Orthopedics, Vydehi Institute of Medical Sciences and Research Centre, Bangalore, IND; 3 Orthopedic Surgery, Vydehi Institute of Medical Sciences and Research Centre, Bangalore, IND

**Keywords:** blood loss, local infiltration, posterior lumbar fusion, spine surgery, tranexamic acid

## Abstract

Introduction

Posterior instrumented lumbar fusion (PLIF) is a commonly performed surgical procedure for degenerative lumbar spine disorders such as lumbar disc herniation, spinal stenosis, and spondylolisthesis. However, the procedure is frequently associated with significant intraoperative and postoperative blood loss due to extensive soft tissue dissection, exposure of cancellous bone, and injury to the epidural venous plexus. Excessive blood loss may result in postoperative anemia, increased transfusion requirements, delayed mobilization, prolonged hospital stay, and increased morbidity. Tranexamic acid (TXA), a synthetic antifibrinolytic agent, has been widely used to reduce blood loss in orthopedic and spine surgeries. This study aimed to evaluate the effectiveness of preoperative local infiltration of TXA in reducing perioperative blood loss in posterior lumbar fusion surgery.

Methodology

This prospective controlled study was conducted in the Department of Orthopedics, Vydehi Institute of Medical Sciences and Research Centre, Bengaluru, from March 2023 to June 2024. A total of 40 patients with degenerative lumbar spine disorders planned for 2-3 level posterior lumbar fixation with or without interbody fusion were included and randomly allocated into two groups. Group 1 (n=20) received 1 g of TXA by preoperative local infiltration bilaterally into the paraspinal muscles before skin incision, while Group 2 (n=20) served as the control group without TXA infiltration. Demographic characteristics, preoperative hematological parameters, intraoperative blood loss, postoperative drain output on postoperative days (PODs) 1, 2, and 3, and postoperative hemoglobin and packed cell volume (PCV) were recorded. Statistical analysis was performed using SPSS version 25.0 (IBM Corp., Armonk, NY, USA), with p<0.05 considered statistically significant.

Results

Baseline demographic and preoperative hematological parameters were comparable between the groups. Although intraoperative blood loss was lower in the TXA group, the difference was not statistically significant. Postoperative drain output was significantly reduced in the TXA group on PODs 2 and 3 (p=0.006 and p<0.001, respectively). POD 3 hemoglobin and PCV were significantly higher in the TXA group (12.74±0.87 g/dL and 37.44±0.86%) compared to the control group (11.33±0.80 g/dL and 34.84±1.66%, respectively), with p<0.001 for both. The reduction in hemoglobin and PCV was significantly less in the TXA group.

Conclusion

Preoperative local infiltration of TXA is an effective and safe method for reducing postoperative blood loss and preserving postoperative hematological parameters in posterior instrumented lumbar fusion surgery. This technique may reduce transfusion requirements and improve postoperative recovery. Larger multicentric studies are recommended for further validation.

## Introduction

Degenerative lumbar spine disorders, including lumbar disc prolapse, lumbar canal stenosis, and degenerative spondylolisthesis, are among the most common causes of chronic low back pain, radiculopathy, and functional disability in the adult and elderly population. When conservative management fails, surgical intervention becomes necessary, and posterior lumbar interbody fusion (PLIF) remains one of the most commonly performed procedures for achieving neural decompression, spinal stabilization, and restoration of spinal alignment. Posterior lumbar fusion offers the advantages of rigid fixation, maintenance of intervertebral height, and improved fusion rates, thereby providing significant symptomatic relief and functional improvement in appropriately selected patients. However, despite its clinical efficacy, PLIF is frequently associated with substantial perioperative blood loss due to extensive paraspinal muscle dissection, prolonged operative time, decortication of cancellous bone surfaces, and injury to the epidural venous plexus, which is particularly fragile and prone to bleeding [1,2]. Perioperative blood loss in lumbar fusion surgery is clinically significant and may lead to postoperative anemia, increased allogeneic blood transfusion requirements, prolonged hospital stay, delayed mobilization, increased risk of infection, and higher perioperative morbidity, especially in elderly patients and those with comorbidities. Previous studies have reported average blood loss of over 800 mL in non-instrumented lumbar fusion surgeries and approximately 1500 mL in instrumented lumbar fusion surgeries. In addition to visible blood loss, hidden blood loss has also been recognized as a major contributor to postoperative hemoglobin decline in spinal surgery and is often underestimated in routine clinical practice [2,3].

Blood transfusion, although effective in restoring circulating volume and oxygen-carrying capacity, is associated with recognized risks including transfusion reactions, immunological complications, transmission of infections, fluid overload, and increased healthcare costs. Therefore, perioperative blood conservation has become an integral component of modern spine surgery practice. Several strategies have been adopted to minimize blood loss, including controlled hypotensive anesthesia, cell salvage systems, autologous transfusion techniques, and pharmacological antifibrinolytic agents. Among these, tranexamic acid (TXA) has emerged as one of the most widely used and effective blood conservation agents in orthopedic surgery [4]. TXA is a synthetic derivative of the amino acid lysine and acts as a potent antifibrinolytic agent by reversibly blocking lysine-binding sites on plasminogen molecules, thereby preventing the conversion of plasminogen to plasmin and inhibiting fibrin clot degradation. By stabilizing the fibrin matrix and preventing premature clot dissolution, TXA effectively reduces both intraoperative and postoperative bleeding. Its efficacy has been well established in total joint arthroplasty, trauma surgery, cardiac surgery, and spine surgery [4,5].

Multiple systematic reviews and meta-analyses have demonstrated that TXA significantly reduces intraoperative blood loss, total blood loss, postoperative drainage volume, and blood transfusion rates in spine surgeries without a significant increase in reported thromboembolic complications. Recent evidence specifically in lumbar fusion procedures has shown that TXA reduces intraoperative blood loss, postoperative drain output, and overall transfusion requirements, making it an important adjunct in perioperative blood management [5,6]. Although intravenous administration of TXA has been the most extensively studied and commonly used route, concerns regarding systemic adverse effects and the theoretical risk of thromboembolic complications have generated increasing interest in topical and local administration routes. Local infiltration of TXA directly into the paraspinal muscles before incision is hypothesized to offer the theoretical advantage of achieving high local tissue concentration at the surgical site with minimal systemic exposure. This may provide effective local hemostasis, reduce postoperative drain output, and preserve postoperative hemoglobin levels while minimizing systemic adverse effects. However, compared with intravenous and topical administration, literature regarding the efficacy of preoperative local infiltration of TXA in posterior lumbar fusion surgery remains limited [6,7].

In view of the limited evidence regarding this route of administration, the present study was undertaken to evaluate the efficacy of preoperative local infiltration of TXA in reducing perioperative blood loss in posterior lumbar fusion surgery, with particular emphasis on intraoperative blood loss, postoperative drain output, and hematological parameters.

## Materials and methods

Study design and setting

This study was designed as a prospective controlled study conducted in the Department of Orthopedics, Vydehi Institute of Medical Sciences and Research Centre, Bengaluru, over a period of 18 months from March 2023 to August 2024. A prospective design was chosen to ensure standardized patient recruitment, uniform surgical technique, prospective documentation of perioperative variables, and accurate assessment of blood loss-related outcomes. Similar prospective randomized and controlled methodologies have previously been employed in studies evaluating the efficacy of TXA in spine surgeries [5,8]. The study included patients aged more than 18 years who were diagnosed with degenerative lumbar spine disorders, including lumbar disc prolapse, lumbar canal stenosis, and degenerative spondylolisthesis, and who were planned for two to three-level posterior lumbar fixation with or without PLIF.

Inclusion and exclusion criteria

Patients fulfilling the inclusion criteria were enrolled in the study. The inclusion criteria comprised age ≥18 years, body mass index between 18.5 and 24.9 kg/m², diagnosed degenerative lumbar spine pathology requiring surgical intervention, planned two to three-level posterior lumbar fixation with or without interbody fusion, and willingness to provide written informed consent.

Patients were excluded if they had preoperative hemoglobin <10 g/dL in females or <11 g/dL in males, platelet count <100,000/mm³, known coagulopathy or bleeding disorders, INR >1.4, prolonged activated partial thromboplastin time (>1.4 times normal), current use of aspirin, clopidogrel, or nonsteroidal anti-inflammatory drugs within one week prior to surgery, hepatic, renal, or cardiopulmonary dysfunction, or if they were medically unfit for surgery. These criteria were selected to minimize confounding factors that could independently influence perioperative bleeding and postoperative hematological outcomes [9].

Surgical procedure and data collection

All procedures were performed by the same surgical team under general anesthesia using a standardized posterior approach to the lumbar spine. Patients were positioned prone on a radiolucent table. Following sterile preparation and draping, a midline posterior incision was made, followed by subperiosteal dissection of the paraspinal muscles to expose the posterior spinal elements. In the study group, 1 g of TXA diluted in an appropriate volume was infiltrated bilaterally into the paraspinal musculature before skin incision. This timing was chosen to allow adequate local tissue saturation before surgical dissection [8]. Posterior pedicle screw fixation with two to three-level lumbar fusion with or without PLIF was performed. Standard decompression procedures, including laminectomy, facetectomy, and bilateral foraminotomy, were carried out wherever indicated. Interbody cages and bone grafting were used as required based on the pathology and intraoperative findings. A closed vacuum suction drain was placed deep to the fascia before wound closure in all patients [10]. The perioperative data collected included demographic details, preoperative hematological parameters, intraoperative blood loss, duration of surgery, number of fusion levels, postoperative drain output at 24, 48, and 72 hours, and postoperative hemoglobin and packed cell volume (PCV) on postoperative day (POD) 3.

Intraoperative blood loss was estimated using the following formula: Estimated blood loss (EBL) (mL)=(W_soaked_−W_dry_)+(V_suction_−V_irrigation_), where W_soaked_ represents the weight of soaked surgical sponges (g), W_dry_ represents the weight of dry surgical sponges (g), V_suction_ represents the total volume collected in the suction canister (mL), and V_irrigation_ represents the total irrigation fluid used intraoperatively (mL). In other words, estimated blood loss was determined as the sum of blood absorbed by surgical sponges and blood collected in the suction canister after subtracting the irrigation fluid used.

Since 1 g of blood is approximately equivalent to 1 mL, the sponge weight difference was directly expressed in milliliters, and this method has been used in previous spine surgery studies [1,8]. Postoperative blood loss was assessed by measuring drain output at 24-hour intervals up to POD 3. The primary outcome measure was perioperative blood loss, assessed as intraoperative blood loss and postoperative drain output. Secondary outcome measures included postoperative hemoglobin and PCV levels, as well as the reduction in these parameters from the preoperative period to POD 3.

Sample size and sampling technique

The sample size was calculated based on the findings of a previously published study by Arun-Kumar and Naresh-Babu [8], which reported a mean intraoperative blood loss of 223.6±40.1 mL in the TXA group and 344.0±88.5 mL in the control group. Using these values, with a 95% confidence interval (α=0.05) and 80% study power (β=0.20), the minimum required sample size was estimated to be six patients in each group, resulting in a total sample size of 12 patients. However, to improve the statistical validity, enhance the power of the study, and increase the reliability and generalizability of the results, a total of 40 patients were included in the present study, with 20 patients in each group. Patients were recruited by consecutive sampling after applying the inclusion and exclusion criteria and were subsequently randomized into study groups. The patients were randomly allocated into two groups. Group 1 (TXA group, n=20) received 1 g of TXA by preoperative local infiltration bilaterally into the paraspinal muscles prior to skin incision, whereas Group 2 (control group, n = 20) underwent the same surgical procedure without TXA infiltration. The local infiltration route was selected based on previously described methods demonstrating effective local hemostasis with minimal systemic exposure [8]. The collected data were entered into Microsoft Excel and analyzed using SPSS version 25.0 (IBM Corp., Armonk, NY, USA). Qualitative variables were expressed as frequencies and percentages, while quantitative variables were expressed as mean ± standard deviation. Normality of data distribution was assessed using the Shapiro-Wilk test. Intergroup comparison of continuous variables was performed using the independent samples t-test, whereas intragroup comparison of preoperative and postoperative values was performed using the paired t-test. Nonparametric tests such as the Wilcoxon signed-rank test were used wherever applicable. A p-value <0.05 was considered statistically significant [5,11].

## Results

A total of 40 patients undergoing posterior instrumented lumbar fusion surgery for degenerative lumbar spine disorders were included in the study. The patients were equally distributed into two groups, with 20 patients in the TXA group and 20 patients in the control group. Both groups were comparable at baseline with respect to demographic characteristics and preoperative hematological parameters, ensuring adequate homogeneity for comparison. The mean age of patients in the TXA group was 52.4±8.6 years, while that in the control group was 53.1±7.9 years, with no statistically significant difference (p=0.78). Gender distribution and body mass index were also comparable between the two groups (p>0.05). Similarly, preoperative hematological parameters, including hemoglobin, PCV, prothrombin time, activated partial thromboplastin time, and INR, did not differ significantly between the two groups, indicating similar preoperative status (Table 1).

**Table 1:** Baseline demographic and preoperative hematological characteristics Continuous variables were compared using the independent samples t-test, and categorical variables were compared using the chi-square test. Hb: hemoglobin; PCV: packed cell volume; PT: prothrombin time; APTT: activated partial thromboplastin time; INR: international normalized ratio; TXA: tranexamic acid

Variable	TXA group (n=20)	Control group (n=20)	Test statistic	P-value
Age (years)	52.4±8.6	53.1±7.9	t=0.27	0.78
Male, n (%)	11 (55)	10 (50)	χ²=0.10	0.75
Female, n (%)	9 (45)	10 (50)	χ²=0.10	0.75
BMI (kg/m²)	23.2±1.4	23.5±1.6	t=0.61	0.54
Hb (g/dL)	13.58±0.94	13.49±0.88	t=0.33	0.74
PCV (%)	39.86±1.22	39.44±1.35	t=1.03	0.31
PT (sec)	12.1±0.6	12.3±0.7	t=0.81	0.42
APTT (sec)	31.2±1.5	31.6±1.8	t=0.71	0.48
INR	1.02±0.04	1.03±0.05	t=0.59	0.56

Intraoperatively, the mean blood loss was lower in the TXA group compared to the control group, with values of 421.6±72.4 mL and 435.8±81.2 mL, respectively. However, this difference was not statistically significant (p=0.821). This suggests that although local TXA infiltration may have contributed to an overall reduction in bleeding, its principal benefit may not lie in the control of intraoperative blood loss. Postoperatively, a significant reduction in drain output was observed in the TXA group, particularly on POD 2 and POD 3. On POD 1, the difference in drain output between the groups was not statistically significant. However, by POD 2, drain output was significantly lower in the TXA group (76.4±14.2 mL) compared to the control group (102.8±18.7 mL), with a p-value of 0.006. On POD 3, this difference became even more pronounced, with the TXA group showing 28.6±8.4 mL compared to 56.2±10.5 mL in the control group (p<0.001) (Table 2).

**Table 2:** Intraoperative blood loss and postoperative drain output Continuous variables were compared using the independent samples t-test. TXA: tranexamic acid; POD: postoperative day

Parameter	TXA group	Control group	Test statistic	P-value
Intraoperative blood loss (mL)	421.6±72.4	435.8±81.2	t=0.23	0.821
Drain POD 1 (mL)	148.5±20.3	156.7±24.1	t=1.19	0.243
Drain POD 2 (mL)	76.4±14.2	102.8±18.7	t=2.96	0.006
Drain POD 3 (mL)	28.6±8.4	56.2±10.5	t=4.72	<0.001

Postoperative hematological analysis revealed significantly better preservation of hemoglobin and PCV in the TXA group. On POD 3, the mean hemoglobin in the TXA group was 12.74±0.87 g/dL, whereas in the control group it was 11.33±0.80 g/dL, which was highly significant (p<0.001). Similarly, PCV was significantly higher in the TXA group (37.44±0.86%) compared to the control group (34.84±1.66%), with p<0.001. The mean reduction in hemoglobin from the preoperative period to POD 3 was 0.84±0.22 g/dL in the TXA group and 2.16±0.36 g/dL in the control group, which was statistically significant (p<0.001). A similar significant difference was observed in the reduction in PCV (Table 3).

**Table 3:** Postoperative hematological outcomes Continuous variables were compared using the independent samples t-test. POD: postoperative day; PCV: packed cell volume; Hb: hemoglobin; TXA: tranexamic acid

Parameter	TXA group	Control group	Test statistic	P-value
Hb POD 3 (g/dL)	12.74±0.87	11.33±0.80	t=5.32	<0.001
PCV POD3 (%)	37.44±0.86	34.84±1.66	t=6.01	<0.001
Hb reduction (g/dL)	0.84±0.22	2.16±0.36	t=7.84	<0.001
PCV reduction (%)	2.42±0.64	4.60±0.91	t=6.88	<0.001

Overall, the study findings showed that preoperative local infiltration of TXA significantly reduced postoperative blood loss and preserved postoperative hematological parameters, although the reduction in intraoperative blood loss was not statistically significant.

## Discussion

The present prospective controlled study evaluated the efficacy of preoperative local infiltration of TXA in reducing perioperative blood loss in posterior lumbar fusion surgery. The principal findings of this study were that local TXA infiltration significantly reduced postoperative drain output, preserved postoperative hemoglobin and PCV, and resulted in a significantly lower decline in hematological parameters compared to the control group. However, the reduction in intraoperative blood loss did not reach statistical significance. Lumbar fusion surgeries, particularly multilevel posterior instrumented procedures, are known to be associated with substantial blood loss due to extensive muscle dissection, decortication of cancellous bone surfaces, prolonged operative time, and injury to the epidural venous plexus [1]. In the present study, although the mean intraoperative blood loss was lower in the TXA group, the difference was not statistically significant. This finding is consistent with the study by Arun-Kumar and Naresh-Babu, who reported that local infiltration of TXA showed a trend toward reduced intraoperative blood loss but with comparable effectiveness to intravenous TXA in spine surgeries [8]. A possible explanation is that local infiltration primarily acts at the soft tissue level and may be more effective in controlling postoperative oozing from muscle and subfascial planes than active intraoperative cancellous bone bleeding.

A major finding of the present study was the significant reduction in postoperative drain output on PODs 2 and 3 in the TXA group. This suggests that preoperative local infiltration of TXA may provide sustained local antifibrinolytic action in the paraspinal muscles and wound bed, thereby reducing postoperative microvascular oozing and fibrinolysis-related bleeding. Similar findings were reported by Wang et al., who demonstrated that preoperative single-dose TXA significantly reduced postoperative blood loss in posterior lumbar surgery without major adverse effects [12]. Furthermore, a recent meta-analysis by Luan et al. showed that TXA significantly reduces drainage volume and total blood loss in PLIF surgeries [5]. The findings of our study are therefore in agreement with the growing body of evidence supporting the hemostatic efficacy of TXA in spine surgery. Another important observation in the present study was the significantly higher POD 3 hemoglobin and PCV values in the TXA group. Patients receiving local TXA infiltration had better preservation of postoperative hematological parameters, indicating effective perioperative blood conservation. This result is clinically relevant, as postoperative anemia can delay mobilization, prolong hospital stay, and increase transfusion requirements. Similar observations were reported in the randomized clinical trial by Hao et al., where TXA administration significantly reduced visible and hidden blood loss and preserved postoperative hemoglobin levels in PLIF surgery [10]. Meta-analytic evidence has also consistently shown a significantly lower hemoglobin drop in TXA-treated patients [11].

The significantly lower decline in hemoglobin and PCV observed in our study further reinforces the efficacy of local TXA infiltration. This is likely due to inhibition of local fibrinolysis at the surgical site, leading to stabilization of fibrin clot formation and reduction in postoperative wound bleeding. TXA acts by competitively inhibiting the lysine-binding sites on plasminogen, thereby preventing its conversion to plasmin and reducing fibrin degradation [9]. This pharmacological mechanism may explain the better postoperative hematological preservation seen in our patients. Importantly, our findings also align with the conclusions of multiple recent systematic reviews and meta-analyses. A comprehensive meta-analysis by Xiao et al. demonstrated that TXA significantly lowers postoperative blood loss, total blood loss, drainage volume, transfusion rate, and hemoglobin drop in lumbar surgeries [11]. Similarly, Yamanouchi et al. concluded that TXA significantly reduces intraoperative and total estimated blood loss in spinal surgeries without a significant increase in reported thromboembolic complications [13]. One of the strengths of the present study is the use of local infiltration before incision, which theoretically offers high local tissue concentration with minimal systemic exposure. This approach may reduce theoretical systemic thromboembolic risk associated with intravenous administration, although our study was not powered to detect rare adverse events. Nevertheless, the absence of clinically significant complications in our cohort supports the safety profile of local infiltration TXA.

However, the study has certain limitations. The sample size was relatively small, and the study was conducted at a single center, which may limit the generalizability of the findings. In addition, hidden blood loss was not separately quantified, which may have led to an underestimation of total blood loss. Future multicentric randomized studies with larger sample sizes and comparison with intravenous and topical routes are warranted. Overall, the findings of the present study support preoperative local TXA infiltration as an effective blood conservation strategy in posterior lumbar fusion surgery, particularly in reducing postoperative wound drainage and preserving postoperative hematological status.

## Conclusions

Preoperative local infiltration of tranexamic acid in posterior lumbar fusion surgery was found to be an effective method for reducing postoperative blood loss. Although the reduction in intraoperative blood loss was not statistically significant, the TXA group demonstrated significantly lower postoperative drain output on PODs 2 and 3, along with better preservation of postoperative hemoglobin and PCV compared to the control group. These findings indicate improved local hemostasis and effective perioperative blood conservation. By reducing postoperative anemia and transfusion requirements, this technique may contribute to enhanced postoperative recovery after lumbar fusion surgery. Further large-scale multicentric randomized studies are recommended to validate these findings and establish standardized protocols for their routine clinical use.
